# Fluorimetric Properties of 3-Aminoflavone Biomolecule (3-AF). X-ray Crystal Structure of New Polymorph of 3-AF

**DOI:** 10.3390/molecules24162927

**Published:** 2019-08-13

**Authors:** Wojciech Pająk, Małgorzata Fabijańska, Jakub Wojciechowski, Wojciech M. Wolf, Anna Kilanowicz, Elżbieta Brzezińska, Justyn Ochocki

**Affiliations:** 1 Department of Analytical Chemistry, 1 Muszyńskiego Str., Medical University of Lodz, 90-151 Lodz, Poland; 2 Department of Bioinorganic Chemistry, 1 Muszyńskiego Str., Medical University of Lodz, 90-151 Lodz, Poland; 3 Institute of General and Ecological Chemistry, Łódź University of Technology, 116 Żeromskiego St., 90-924 Lodz, Poland; 4 Department of Toxicology, 1 Muszyńskiego Str., Medical University of Lodz, 90-151 Lodz, Poland

**Keywords:** 3-aminoflavone, X-ray crystallography, fluorescence, LOD (limit of detection), LOQ (limit of quantification), concentration quenching

## Abstract

The crystal structure of the new polymorphic form of 3-aminoflavone (3-AF) has been determined by single crystal X-ray diffraction. This report presents results of fluorimetric studies on 3-AF in methanol and aquatic solvents. Based on 3D fluorescence emission spectra, optimal values for excitation (λ_ex_) and emission/analytical (λ_em_) wavelength, the analytical concentration range as well as the range of concentration quenching for the studied compound were established. Moreover, the limit of detection (LOD) and the limit of quantification (LOQ) were determined. The results were compared with those obtained using the standard UV-Vis absorption spectrophotometric method. The effect of acidity (pH) and the concentration of halide anions (chlorides, bromides, iodides and fluorides) on fluorescence quenching were analysed.

## 1. Introduction

Flavonoids belong to a group of plant secondary metabolites that display a wide spectrum of biological activity. Chemically, flavonoids have the general structure of a 15-carbon skeleton, which consists of two phenyl rings and a heterocyclic ring. The compounds are derivatives of benzo-γ-pyrone. The biological activity, metabolism, chemical and physical properties of flavonoids depend on the kind, number and position of the functional groups attached to the molecule. Natural flavonoids as well as their synthetic derivatives display various pharmacological activities, including anti-oxidative, anti-inflammatory, vasoprotective and anti-proliferative. This class of compounds has been a major object of scientific interest since the discovery of their anti-oxidative activity, i.e., the ability to scavenge free radicals and reactive oxygen species (ROS) [1,2,3,4,5].

Flavonoid derivatives, particularly complexes containing metals, also demonstrate biological and pharmacological properties. Their derivatives that could be applied in antineoplastic chemotherapy are particularly interesting [6]. For this purpose, among others, 3-aminoflavone complexes were synthesized [7,8,9,10,11,12,13]. The trans-Pt_2_Cl_2_ complex (referred to as TCAP) is an example of such synthesis. The compound appeared to be cytotoxic for many tested cancer cell lines. Besides that, it proved to be much less toxic for normal lymphocytes in comparison to cisplatinum, which decreases the number of potential adverse effects. Therefore, it can be a potential drug in antineoplastic therapy if cisplatinum cannot be administered. 

Ruthenium(III) is another metal which forms interesting complexes with 3-aminoflavone. In vitro and in vivo studies revealed good antineoplastic properties of this complex. Some ruthenium complexes, despite their low toxicity, demonstrate effective proapoptotic or antimetastatic properties. Thus, ruthenium complexes with flavonoid compounds could constitute an interesting group of compounds that have potential antineoplastic activity [14]. 

The ability of flavonoids to coordinate metal ions is also applied in the improvement of preparative and analytical techniques. Because of their structure and electron configuration, a great number of flavonoid compounds display fluorescence after excitation with visible or UV light. The structure of rings with a system of conjugated double bonds and delocalized π electrons makes the molecules highly sensitive colorimetric and fluorimetric reagents in analytical chemistry. Chelation of metal ions often affects or quenches the fluorescence in a specific way that can be measured and applied for the quantitative determination of flavonoid-metal complexes [6,15,16]. Furthermore, this method could be finally applied to the detection of potential drugs.

M. Rhia L. Stone et al. reported and discussed fluorescent antibiotics as new tools to fight bacteria resistance. Applications of fluorescent antibiotics include: Examining toxicity and mode of action of the drug, detecting the presence of antibiotics and diagnostic imaging of bacterial infection [17].

**Figure 1:**
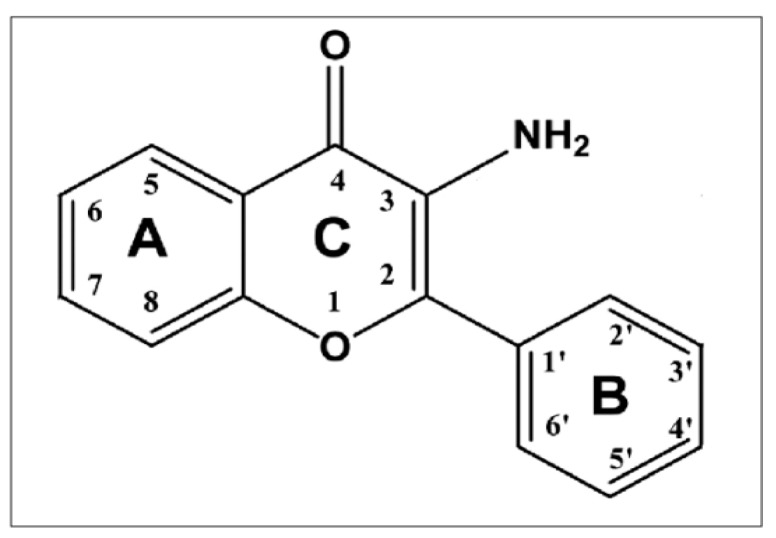
Structural formula of 3-aminoflavone.

As a continuation of our study on metal complexes with flavone derivatives, a novel polymorph of 3-aminoflavone (Figure 1) was discovered, and its crystal structure as well as detailed fluorimetric study was described. 

## 2. Results and Discussion

### 2.1. Crystal Structure Studies

**Figure 2:**
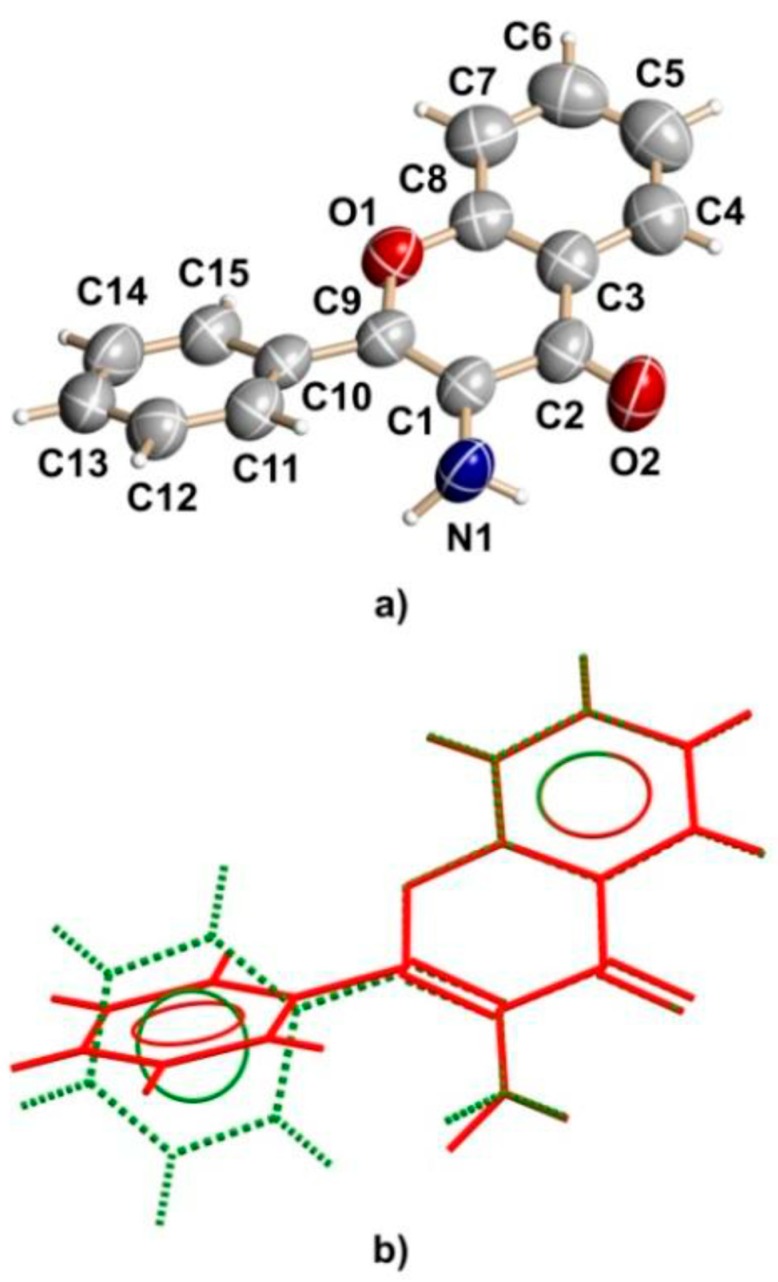
A view of **1o** with atom numbering scheme. Displacement ellipsoids for non-hydrogen atoms were draw with 50% probability level—(**a**); superposition of 3-AF molecules from crystal **1o** (red) and centrosymmetric **1m** (dashed green)—(**b**).

The molecular structure of a novel orthorhombic polymorph of 3-AF, i.e., **1o**, is presented in Figure 2a. Its superposition on the already reported monoclinic structure [13] **1m** is shown in Figure 2b. The former crystallizes in a chiral P2_1_2_1_2_1_ space group while crystals of the latter adopt the centrosymmetric P2_1_/n symmetry. In both structures the benzopyran moieties are practically planar, root mean *square* (r.m.s.) deviations for **1o** and **1m** are 0.01 and 0.02 Å, respectively. Molecular conformation is restricted by the mutual positions of benzopyran and phenyl fragments and may be conveniently defined by the dihedral angle between both moieties, φ = 28.7(4) and 40.7(5)°, as in **1o** and **1m**, respectively. The exocyclic amine groups adopt diverse configurations in both polymorphs. In **1o** the nitrogen atom adopts the tetrahedral sp^3^ configuration while in **1m** it is planar, indicating the sp^2^ hybridization. Interestingly, none of the amine hydrogens are involved in the regular intermolecular hydrogen bonds. They form close contacts with the neighboring carbonyl oxygens, which may be regarded as intramolecular hydrogen bonds [O^…^N 2.673(4), 2.684(5) Å; O^...^H-N 114(2), 101(2)°; O^…^H 2.21(3), 2.36(2) Å for **1o** and **1m**, respectively. Bond lengths are close to those observed in related compounds. The major difference between both polymorphs is related to the exocyclic carbonyl bonds conjugated with the endocyclic C=C bond. The former is slightly longer in **1m** as compared to that in **1o** (1.244(3) and 1.224(2) Å, respectively), while the latter behaves in the opposite way (1.358(3) and 1.381(2) Å, respectively). This indicates that the following electron delocalization is slightly higher in the reported orthorhombic polymorph **1o** than that in monoclinic **1m**.

**Figure 3:**
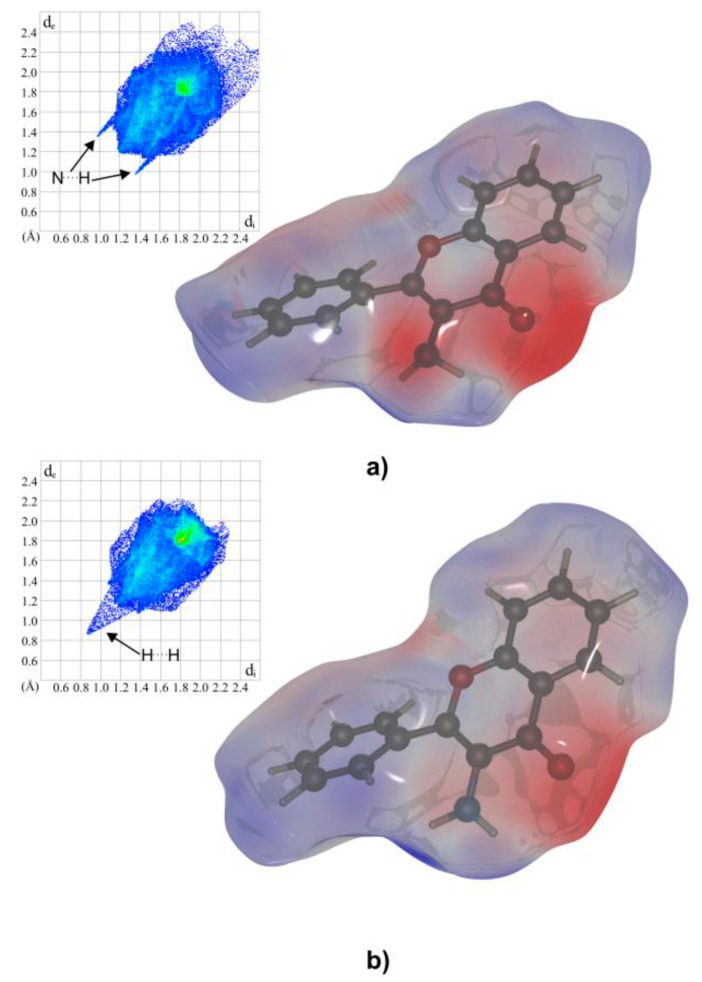
Hirshfeld surfaces mapped with electrostatic potential calculated with B3LYP/6-; 311++G(d,p) and the fingerprint plots for **1o**—(**a**) and **1m**—(**b**).

A CrystalExplorer program [18] was used to investigate differences in the packing and intermolecular interactions between both polymorphs. Hirshfeld surfaces mapped with electrostatic potential calculated at the B3LYP/6- 311++G(d,p) level and fingerprint plots (scattergrams of external (de) and internal (di) contact distance from atoms to Hirshfeld surface) for **1m** and **1o** are presented in Figure 3. The main difference is visualized as a negative charge above the amine group in **1o**. It can be assigned to a lone pair localized on the sp^3^ hybridized nitrogen atom. 

In both polymorphs, weak intermolecular interaction between amine groups can be observed. In **1m** it is a short (1.86(2) Å) H^...^H contact, while in **1o** it is a strong contact H^...^N (2.36(2) Å). The latter stabilizes infinite chains along the [100] twofold screw axis, which generates a helical arrangement of molecules. The search made with the CCDC Mercury program in CSD version 5.36 revealed 297 identical motifs created by the –NH_2_ group with the average N^...^H contact distance 2.450 Å. Similar weak stacking interactions between benzopyrane moieties can be found in both polymorphs (distances between respective ring centroids are 3.586(2) Å and 3.661(2) Å in **1m** and **1o**, respectively).

### 2.2. Electron Absorption Spectra (UV-Vis) of 3-Aminoflavone in Water and Methanol

In preliminary studies, spectra of 3-AF were recorded in water and methanol. The studies were aimed at estimating the solubility of the compound and checking its stability in solution. 

(a) Absorption spectroscopy studies in methanol 

**Figure 4:**
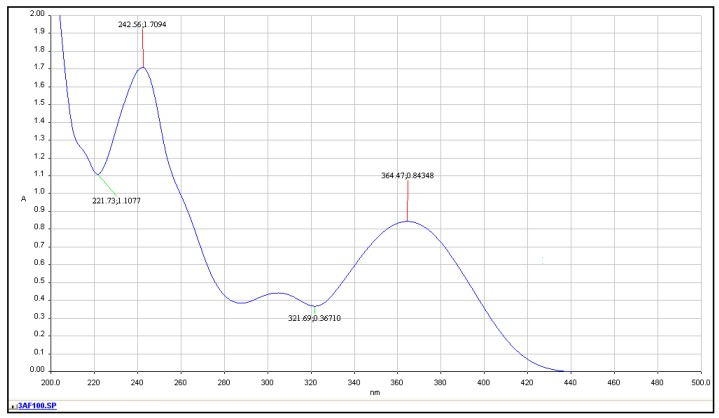
UV-Vis absorption spectrum of 3-aminoflavone in methanol at a concentration of 100 μg/mL, in quartz cuvette with l = 0.2 cm, at temperature t = 20 °C, with methanol as the reference.

It was established by us that the solubility of the studied compound is much better in methanol than in water. Figure 4 presents the UV-Vis spectrum of 3-aminoflavone, at a concentration of c = 100 μg/mL, which corresponds to a molar concentration c_m_ = 0.1 g/L(237.252 g/mol) = 4.215 · 10^−4^ mol/L.

The observed spectrum has three distinctive bands with maxima at λ = 364, 305 and 243 nm with molar absorption coefficients 10,005, 5220 and 20,277, respectively. It was confirmed that the spectra are stable over time for at least a few days.

(b) Absorption Spectroscopy Studies in Water

The observed UV-Vis spectrum of 3-AF in water also has two distinct bands with maxima at λ = 352 nm and λ = 243 nm. A comparison of this spectrum with the 3-AF spectrum in methanol confirms general similarities between these two spectra. A change in the position of the long-wave band with the maximum at λ = 352 nm can be observed, i.e., a hypsochromic shift of 12 nm (in methanol, the position was λ = 364 nm). Moreover, ex tempore studies revealed that 3-AF is poorly water soluble. It was estimated that at room temperature, solubility of 3-AF is not higher than 8 μg/mL. Thus, further fluorimetric studies were conducted in methanol, in which this compound dissolves relatively easily. 

### 2.3. Three-Dimensional Fluorescence Spectra of 3-Aminoflavone in Methanol 

**Figure 5:**
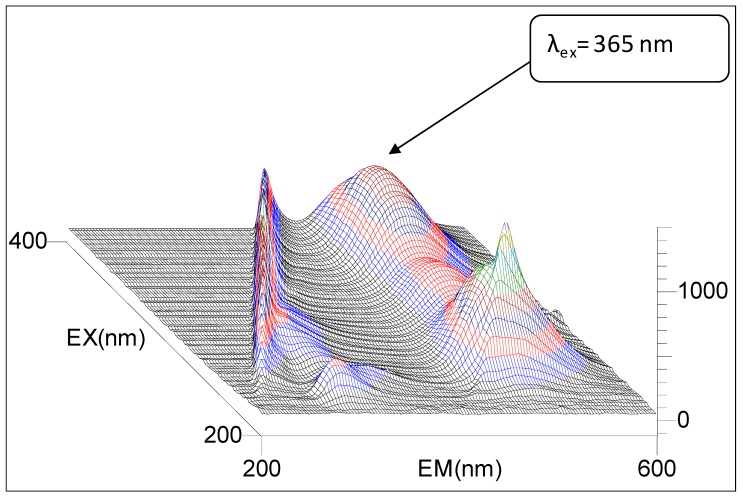
Three-dimensional spectrum of 3-aminoflavone in methanol. Intensity of fluorescence emission I_f_ in RFU (relative fluorescence unit). Instrument parameters. Measurement type: 3D scan; data mode: Fluorescence; EX (Excitation) sampling interval: 3.0 nm; EM (Emission) sampling interval: 3.0 nm; scan speed: 1200 nm/min; EX slit: 5.0 nm; EM slit: 5.0 nm; PMT (Photomultiplier Tube) voltage: 700 V; sensitivity: 1; threshold: 1.0.

For the purpose of fluorimetric quantitative determination, it is necessary to calculate a pair of electromagnetic radiation wavelengths, for which fluorescence emission signal is maximal. The first is the wavelength of excitation fluorescence λ_ex_ and the other is the wavelength of emission fluorescence λ_em_. These values are read at the maximum of the strongest emission band, performed using the 3D technique. Figure 5 presents a 3D spectrum of 3-aminoflavone in methanol, with marked λ_ex_ and λ_em_ of the strongest emission band, measured with the use of Hitachi F4500 spectrofluorimeter. These wavelengths allowed for proper selection of filters for quantitative fluorescence measurements on a Jenway 6285 fluorimeter. 

**Figure 6:**
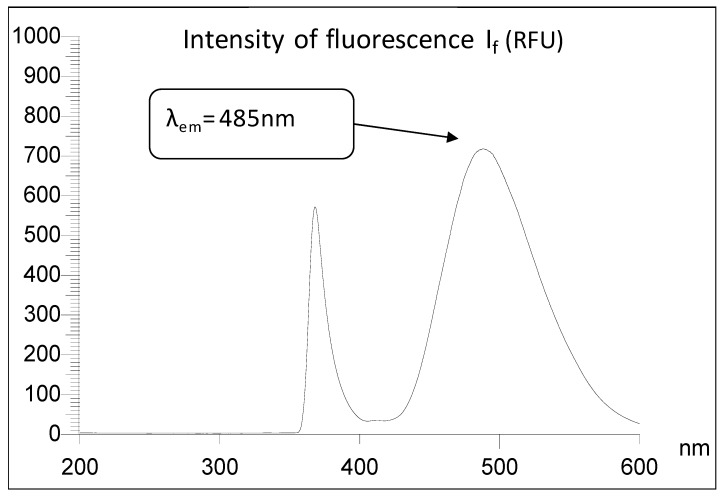
Emission spectrum of 3-aminoflavone in methanol at a concentration c = 0.1 μg/mL in quartz cuvette with a thickness l = 1 cm. Fluorescence excitation λ_ex_ = 365 nm; intensity of fluorescence emission I_f_ is presented in RFU (relative fluorescence unit).

It is easy to infer from the spectrum that the strongest fluorescence emission is observed for λ_ex_ = 365 nm (excitation filter) and for λ_em_ = 485 nm (analytical filter). These values are coordinates of the maximum of the strongest emission peak (Figure 5). The emission spectrum of 3-AF for the best excitation wavelength λ_ex_ = 365 nm is presented in Figure 6.

### 2.4. Determination of Limit of Detection (LOD) and Quantification (LOQ) of 3-Aminoflavone by the Fluorimetric Method

The limit of detection and the limit of quantification are basic parameters in the process of validation of analytical methods [19]. In order to determine LOD and LOQ, adopting linear dependence y = (bx + a) (y and x are dependent and independent variable, respectively), we should calculate direction coefficient b, absolute term a, errors of these coefficients S_b_, S_a_ and residual deviation S_y/x_. These values are calculated according to the following formulas [19]:

$$b=\frac{n\Sigma \;x_{i}y_{i}-\Sigma \;x_{i}\Sigma \;y_{i}}{n\Sigma \;x_{i}{}^{2}-{\left(\Sigma \;x_{i}\right)}^{2}}\;a=\frac{1}{n}\;\left(\Sigma \;y_{i}-b\Sigma \;x_{i}\right)$$

$$S_{b}=\sqrt{\frac{n\;\left(\Sigma \;y_{i}{}^{2}-b\Sigma \;x_{i}y_{i}-a\Sigma \;y_{i}\right)}{\left(n-2\right)\;{\left[\;n\Sigma \;x_{i}{}^{2}-{\left(\Sigma \;x_{i}{}^{}\right)}^{2}\right]}^{}}}\;S_{a}=\sqrt{\frac{1}{n}S_{b}{}^{2}\;\Sigma \;x_{i}{}^{2}}$$

$$S_{y/x}=\sqrt{\frac{\Sigma {\left(y_{i}-{\overset{\wedge }{y}}_{i}\right)}^{2}}{\left(n-2\right)\;}}\;\mathrm{where}\;{\overset{\wedge }{y}}_{i}=a\cdot x_{i}+b$$

LOD can be calculated with two different methods, according to the formulas given below [20,21,22]:

a. method I: LOD = 3.3 · S_a_ /b

b. method II: LOD = 3.3 · S_y/x_ /b

LOQ is a multiple of LOD, calculated according to the formula: LOQ = 3 · LOD.

In order to determine LOD and LOQ with the application of the fluorimetric method, a Jenway 6285 fluorimeter was used, which was equipped with a set of light filters for the excitation and emission beam. Filters were selected for transmission corresponding to the wavelengths previously determined using the 3D spectrum. Thus, the excitation filter λ_ex_ was selected with transmission at a wavelength of 350 nm, whereas the analytical filter λ_em_ = 470 nm. 

**Figure 7:**
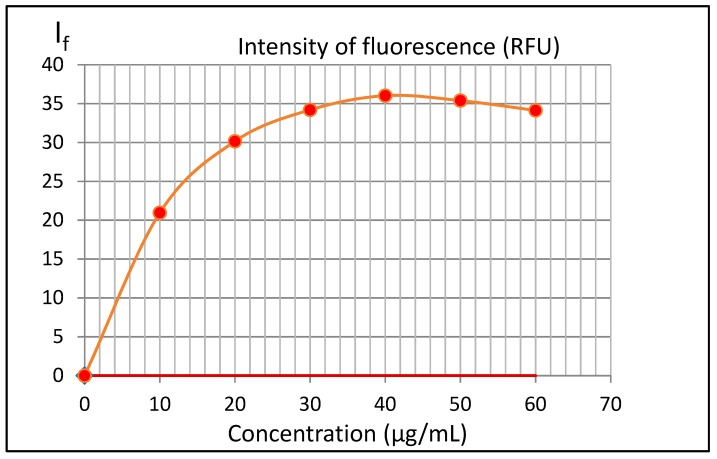
Intensity of fluorescence emission I_f_ as a function of 3-aminoflavone concentration in methanol. The fluorescence was measured in cuvette with a path length l of 1 cm. For excitation and emission, filters with wavelengths of 350 nm and 470 nm were used, respectively.

In the preliminary experiment, the concentration range of 3-AF was determined, in which I_f_ was a linear function of the concentration, by measuring emission spectra of a series of six solutions in methanol with a concentration ranging from 10 μg/mL to 60 μg/mL, in steps of 10 μg/mL—see Figure 7.

The above plot clearly shows the effect of concentration quenching. For concentrations above 10 μg/mL, the I_f_ value is no longer linear and for concentrations above 40 μg/mL, due to further concentration quenching, fluorescence emission I_f_ even decreases.

**Figure 8:**
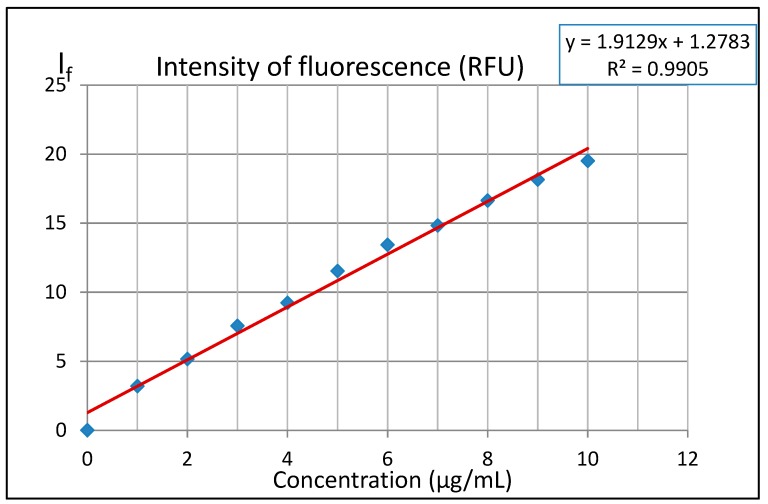
Fluorescence emission intensity dependence as a function of 3-aminoflavone concentration in methanol fitted with a linear function. The cuvette thickness l was 1 cm. For excitation and emission, filters with wavelengths of 350 nm and 470 nm were used, respectively.

In order to determine the LOD and LOQ values, a series of ten solutions of 3-AF in methanol was analyzed, ranging from 1 μg/mL to 10 μg/mL, in steps of 1 μg/mL. Figure 8 presents fluorescence intensity I_f_ measurements as the function of 3-AF concentration.

In the presented study, linear regression was used (EXCEL 2010, REGLINP function). In order to determine LOD and LOQ, direction coefficient b, absolute term a, corresponding error S_a_ and the coefficient of determination R^2^ were calculated. For the purpose of the alternative method of determining LOD and LOQ, S_y/x_ was also calculated. The calculated coefficients b and a and corresponding errors S_a_ and S_y/x_ are the following:

b = 1.9129 ± 0.06258; a = 1.2783 ± 0.3702; S_a_ = 0.3702; S_y/x_ = 0.6563

The value of the calculated coefficient of determination R^2^ = 0.9905 means that the adopted linear model is well adjusted to experimental results. Considering the above results, one can determine LOD and LOQ values with two methods. 

Method I with the use of S_a_: For b = 1.9129 and S_a_ = 0.3702 allows to obtain:

LOD = 3.3 · S_a_/b ≈ 0.64µg/mL; LOQ = 3 · LOD ≈ 1.92 µg/mL

Method II with the use of **S_y/x_**: For b = 1.9129 and S_y/x_ = 0.6563 allows to obtain:

LOD = 3.3 · S_y/x_/b ≈ 1.13 µg/mL; LOQ = 3 · LOD ≈ 3.40 µg/mL

### 2.5. Determination of Limit of Detection (LOD) and Limit of Quantification (LOQ) of 3-Aminoflavone with UV-Vis Spectroscopy

**Figure 9:**
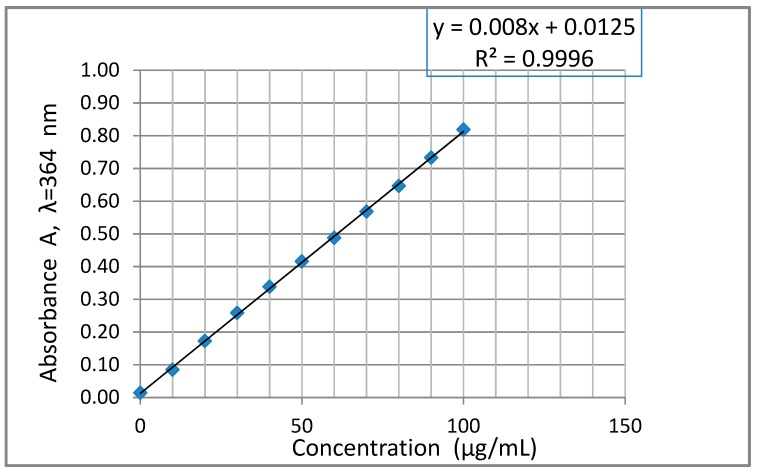
Dependence of absorbance A at λ = 364 nm as the function of 3-aminoflavone concentration in methanol. The quartz cuvette thickness l was 0.2 cm.

In order to determine LOD and LOQ of the 3-AF compound with the UV-Vis spectroscopic method, a series of solutions of 3-AF in methanol, with concentrations ranging from 10 to 100 µg/mL, in steps every 10 µg/mL was prepared. The concentrations were selected in such a way that they allowed to determine the absorbance values to remain within the recommended range of 0.5–2 absorbance units A. Calculations were made for the strongest band, with a maximum at λ = 364 nm. Figure 9 shows the dependence of absorbance A (λ = 364 nm) as a function of 3-AF concentration.

Similar to the previous procedure, direction coefficient b, absolute term a and corresponding errors S_a_ and S_y/x_ were calculated using REGLINP function in the EXCEL spreadsheet. The following values were obtained:

b = 0.00801 ± 0.00005; a = 0.01253 ± 0.00300; S_a_ = 0.00300; S_y/x_ = 0.00533

The calculated coefficient of determination R^2^ = 0.9996 means that the adopted model is well adjusted to experimental results. Considering the above results, as in the fluorimetric calculations, LOD and LOQ values were obtained with two methods. 

Method I with the use of S_a_: For b = 0.00801 and S_a_ = 0.00300 allows to obtain:

LOD = 3.3 · S_a_ /b ≈ 1.24 µg/mL, LOQ = 3 · LOD ≈ 3.71 µg/mL

Method II with the use of S_y/x_: For b = 0.00801 and S_y/x_ = 0.00533 allows to obtain:

LOD = 3.3 · S_y/x_ / b ≈ 2.20 µg/mL; LOQ = 3 · LOD = 3 · 2.20 µg/mL = 6.60 µg/mL

### 2.6. Studies on the Influence of pH on Fluorescence of 3-Aminoflavone Solutions

In order to evaluate the effect of pH on fluorescence of 3-AF, a solution of 3-AF in 5 mL of methanol at a concentration of 10 μg/mL and a water solution of HCl at a concentration 0.2 mol/L were prepared. After dilution, the HCl concentration decreased twofold, to the value of 0.1 mol/L, which results in pH ≈ 1 and 3-AF concentration of 5 μg/mL. For this solution, the 3D fluorescence spectrum was determined as well as optimal values for excitation, i.e., λ_ex_ = 356 nm and emission λ_em_ = 488 nm were calculated. These values are similar to those obtained for methanol solution, i.e., 365 and 485 nm, respectively.

**Figure 10:**
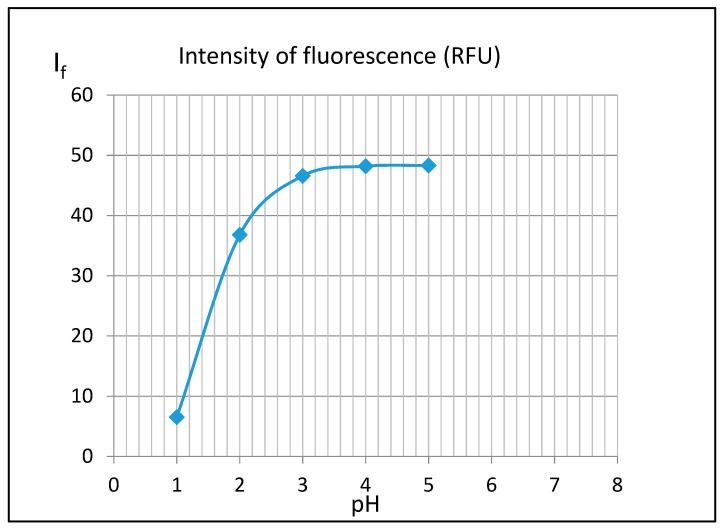
Relationship between fluorescence intensity I_f_ of 3-aminoflavone and pH. For excitation and emission, filters with wavelengths of 350 nm and 470 nm were used, respectively.

Thus, to evaluate the effect of pH on fluorescence of 3-AF, a series of solution spectra of 3-AF in 5 mL of methanol at a concentration of 10 μg/mL and 5 mL of HCl with concentrations of 0.2, 0.02, 0.002, 0.0002 and 0.00002 mol/L were measured. After dilution, pH values of these solutions were around 1, 2, 3, 4 and 5, respectively. Figure 10 presents the relationship between fluorescence intensity I_f_ and pH. 

**Figure 11:**
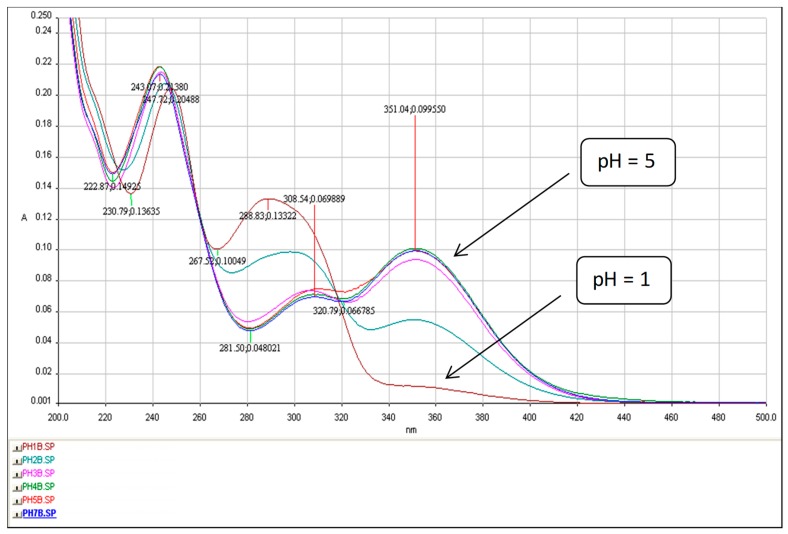
UV-Vis spectra of 3-aminoflavone (c = 5 μg/mL) in HCl solutions at pH = 1, 2, 3, 4, 5. l = 1 cm.

The plot shows that an increase in the acidity of a solution is accompanied by a rapid decrease in 3-AF fluorescence. For pH = 1 it is about 8 times weaker than for pH = 5. Similar experiments were conducted for solutions in which hydrochloric acid was replaced by sulfuric acid. Moreover, here a strong decrease in 3-AF fluorescence was observed. Thus, it can be concluded that fluorescence quenching is caused not by a chloride anion, although the influence of some anions, particularly halide anions, is known, but hydronium ions H_3_O^+^. In order to analyze this phenomenon, UV-Vis spectra of 3-aminoflavone in these solutions were measured— see Figure 11.

The plot clearly shows isosbestic points at λ ≈ 245, 260 and 320 nm. It confirms the presence of acid-base balance (mixture of neutral and protonated forms of 3-AF). Based on this plot, it can be concluded that, for the band with a maximum at λ ≈ 350 nm, an increase in acidity (decreasing pH) is associated with a strong decrease in the absorbance value A. A strong decrease in fluorescence can be explained by the accompanied increase in the acidity of the solutions. The protonated form of 3-AF practically has no absorption band at λ ≈ 350 nm, which is used for fluorescence excitation of the 3-AF molecule.

### 2.7. Influence of Halide Anions on Fluorescence of 3-Aminoflavone

It is generally known that the environment can considerably affect emission spectra of a studied solution considering the position of emission bands and intensities. This influence is particularly strong for solutions containing halide anions. For example, chloride ions are a commonly known quenching agent for quinine [23].

**Figure 12:**
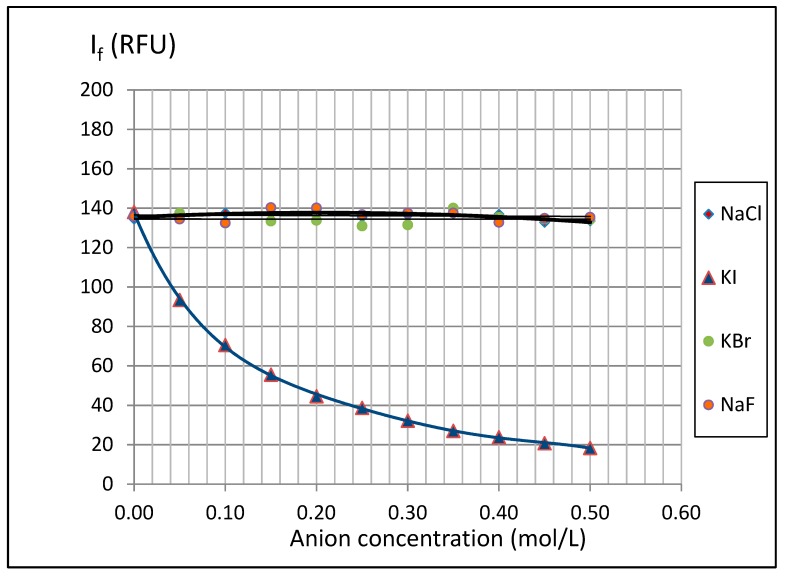
The relationship between fluorescence intensity I_f_ of 3-aminoflavone and **the** concentration of chloride, bromide, iodide and fluoride ions. The concentration of 3-aminoflavone was 5 µg/mL in cuvette with a path length of 1 cm. For excitation and emission, filters with wavelengths of 350 nm and 470 nm were used, respectively.

In this study, an attempt to evaluate an influence of the concentration of these anions on the intensity of 3-AF fluorescence was also made. Mixtures of 3-AF solution in methanol with the corresponding halide anion solutions were prepared. A solution of 3-AF in methanol at a concentration of 10 µg/mL was used as a working solution. Next, to 5 mL of this solution a proper amount of aqueous solution of the particular anion was added. The mixture was filled with water to obtain 10 mL of a halide solution. For example, to prepare the solution with a chloride concentration of 0.1 mol/L, 1 mL of chloride solution with a concentration of 1 mol/L, 5 mL of 3-AF solution and 4 mL of distilled water were mixed to obtain the final 10 mL solution. The concentration of 3-AF in all experiments was always 5 µg/mL, i.e., a double dilution of the 3-AF solution. Results of halide concentration on fluorescence of 3-AF are presented in Figure 12.

It can be observed that the presence of anions such as chloride, bromide and fluoride does not affect the fluorescence of 3-AF. However, strong fluorescence quenching by iodide ions was noted. In solution with iodide anions and concentration c = 0.5 mol/L, fluorescence intensity decreases from 138 RFU to 18 RFU, and this decay is almost eightfold. The mechanism of fluorescence quenching by iodide ions has been described by J. Najbar and M. Mac [24]. According to these authors, the phenomena are associated with the so-called external heavy-atom effect and with transferring the electron from the anion to the excited compound molecule. However, we would like to pay attention to another possible reason for the 3-AF quenching phenomenon. The 1 mol /L KI solution prepared for testing tends to change after a few hours from preparation to a slight yellow colour (oxidation of iodides to free iodine). The UV-Vis spectrum made for this solution has a wide absorption band with a maximum at λ ≈ 352 nm. This is nearly the wavelength of the radiation used to excite 3-AF (λ = 350 nm) and very close to the location of the maximum of the long-term absorption band 3-AF (λ = 364 nm). It should be noted that the concentration of the KI solution (1 mol/L) is greater by several orders of magnitude than that of the examined compound—5 μg/mL (2.11 · 10^−5^ mol /L). The trace amounts of free iodine in the examined solution are therefore a "competitive" source of absorption of the radiation needed to excite the molecule of 3-AF and may be responsible for the fluorescence quenching.

Quantitative determinations of 3-aminoflavone cannot be performed in an aqueous environment because the compound is poorly soluble in water. Hence, in experiments we used methyl alcohol, in which this solubility is sufficient. The fluorimetric method cannot be applied for quantitative determinations if 3-AF concentration exceeds 40 μg/mL. At higher concentrations, the fluorescence intensity I_f_ decreases with increasing 3-AF concentration. We stated that the linear range of fluorescence intensity I_f_ vs concentration of 3-AF is limited to concentrations up to 10 μg/mL. The LOD calculated for this range was 0.64 µg/mL (the method with the use of S_a_) or 1.92 µg/mL (the method with the use of S_y/x_). The LOQ values were 1.13 and 3.40 µg/mL, respectively. 

With regard to spectrophotometric measurements, the range of linear dependence of absorbance A on the concentration of 3-AF is much higher and reaches at least 100 µg/mL. Calculated LOD values were 1.24 µg/mL (the method with the use of S_a_) and 3.71 µg/mL (the method with the use of S_y/x_), respectively. The LOQ values are 2.20 and 6.59 µg/mL, respectively. It can be seen that these values are much higher (“worse”) than those obtained with the application of the fluorimetric method. 

The relationship between pH and 3-AF fluorescence was also analyzed. It is associated with protonation of 3-AF molecules, which manifests itself in clearly visible isosbestic points at λ ≈ 245, 260 and 320 nm. Changes in absorption UV-Vis spectra, related to different pH values, confirm this phenomenon. 

The effect of halide anions on luminescence quenching of 3-AF was analyzed. No significant effect of chloride, bromide or fluoride ions on fluorescence emission was observed. However, a strong fluorescence quenching by iodide ions was noted.

## 3. Materials and Methods

### 3.1. Materials

The 3-aminoflavone compound (IUPAC name: 3-amino-2-phenylchromen-4-one, C_15_H_11_NO_2_, molar mass: 237.258 g/mol; PubChem CID: 248316), referred to as 3-AF, is a flavonoid compound whose structural formula is presented in Figure 1.

The 3-aminoflavone compound was synthesized and purified in our laboratory, and was used as a ligand in many metal complexes. Macroscopically, 3-aminoflavone is yellow and crystalline solid. The crystals are needle shaped, and their melting point ranges between 140 and 142 °C. It is significantly soluble in most organic solvents, but poorly soluble in water. The photophysical and electron properties of 3-AF have been recently reported [25]. The authors described results of theoretical as well as experimental studies. The theoretical calculations were made using the TD DFT model (time-dependent density functional theory). This low-cost calculation method, due to an effective electron correlation, allowed to obtain a reliable description of ground and excited states of the molecule, the transition energies, electron densities and HOMO (highest occupied molecular orbital)/LUMO (lowest unoccupied molecular orbital) energy levels. In this study, an influence of the polarity of solvents on the absorption and emission spectra of 3-AF was also established. The solvent effect is less significant for absorption spectra and more pronounced for fluorescence spectra. For example, the maximum absorption of 3-AF in hexane is observed at the wavelength λ_max_ = 359 nm, while in the case of the aqueous solution, it is at λ_max_ = 353 nm. For fluorescence spectra the maximum emission of 3-AF in hexane is λ_em_ = 425 nm and for the aqueous solution it is λ_em_ = 490 nm. 

It this work, we present the results of 3-AF fluorescence emission in methanol as a function of pH and halogen ion concentration as well as the method for the quantitative determination of 3-AF concentration.

### 3.2. Methods

The data of single crystal X-ray diffraction were collected on a Bruker AXS Smart Apex II CCD diffractometer (Karlsruhe, Germany) with Incoatec IμS Cu-Kα (λ = 1.54178 Å) as a source of radiation. The structure was solved by the direct method and subsequent Fourier syntheses and refined by full-matrix least squares on F2 using the SHELXS-2012 and SHELXL-2012 programs [26]. The scattering factors were those given in the SHELXL program (Goettingen, Germany). All non-hydrogen atoms were refined anisotropically. Hydrogen atoms were generated geometrically and refined as riding atoms with isotropic displacement factors equivalent to 1.2 times those of the atom to which they were attached, except for the two in the amine group, which were allowed to refine freely. Graphics were produced with XShell14 and CCDC Mercury (Cambridge, UK) [27,28].

Crystal data for 1o (3-AF polymorph): C_15_H_11_NO_2_, M = 237.25, orthorhombic, a = 4.1492(4), b = 12.9988(12), c = 21.256(2) Å, V = 1146.46(19) Å^3^, T = 296(5) K, space group P2_1_2_1_2_1_ (no. 19), Z = 4, 12,758 reflections were measured, 2123 were unique (R_int_ = 0.0431) and 2049 were greater than 2σ(F^2^). The final anisotropic full-matrix least-squares refinement on F^2^ with 172 variables converged at R_1_ = 0.0353 for the observed data and *w*R_2_ = 0.1019 for all data. The CCDC deposition number was 1037205.

Fluorescence spectra were measured with the use a Jenway 6285 fluorimeter (spectral range from 190 to 850 nm, Gransmore Green Felsted Dunmow, UK) and Hitachi F4500 spectrofluorimeter (Tokio, Japan), which allows to record complex fluorescence spectra in the 2D and 3D techniques. UV-Vis spectra were registered on a UV-VIS Lambda 25 spectrophotometer (PerkinElmer, Waltham, Massachusetts, USA) in the range from 200 to 800 nm. UV-Vis absorption, as well as fluorescence spectra in the 3D technique, were measured in quartz cuvettes in the full transmission spectral range, λ > 200 nm. For quantitative fluorescence measurements, special plastic cuvettes with transmission λ > 300 nm were used. 

## 4. Conclusions

In conclusion we elaborated a convenient method for quantitative determination of 3-aminoflavone (3-AF). We demonstrated that absorption and emission spectroscopy can be applied for determination of 3-AF. This is important in view of the ability of 3-AF to form coordination compounds with p-, d- and f-electron metal ions, which makes it an interesting component for analytical chemistry. Recently, we reported on the biological activity of the platinum(II) chloride complex of 3-AF as a potential anticancer drug. Our study on 3-aminoflavone could be a basis for further investigation of 3-AF as a powerful tool in a fluorimetric detection of metal complexes or new chemotherapeutics.
